# Spatial Pattern Change and Ecosystem Service Value Dynamics of Ecological and Non-Ecological Redline Areas in Nanjing, China

**DOI:** 10.3390/ijerph18084224

**Published:** 2021-04-16

**Authors:** Minghui Yang, Yu Xie

**Affiliations:** 1 School of Economics and Management, Nanjing Forestry University, Nanjing 210037, China; ymh1730256@163.com; 2 NFU Academy of Chinese Ecological Progress and Forestry Development Studies, Nanjing 210037, China

**Keywords:** Nanjing, ecological conservation red line, production–living–ecological space, ecosystem service valuation

## Abstract

Ecological conservation red line (ECRL) is gaining increasing academic attention as delimiting the minimum space scope of ecological protection and the bottom line of ecological security. Taking Nanjing as a case study, we divided the territory into ecological and non-ecological redline areas (ERAs and NERAs, respectively). This paper highlights two key research issues based on the 2005, 2010, 2015 and 2018 annual remote sensing data: (i) quantitative analysis of the Ecological Redline Policy (ERP) validity by conducting a horizontal comparison of the ERAs and NERAs; and (ii) exploration of the land-use transitions and spatial pattern changes affecting ecosystem service value (ESV). Results showed that delineating ECRL could effectively slow down the decline rate of ESV. The trend of eco-quality deterioration was greater than eco-quality improvement in Nanjing, presenting an ESV that declined slightly in the whole. According to our findings, we suggest that reasonably increasing eco-lands (woodland and water area) and decreasing construction land will enhance the regional ESV. Meanwhile, promoting the transition from production space to ecological space and depressing the encroachment of living space on other space types, will be instrumental in mitigating the ESV decline. The results of this study are expected to provide valuable implications for spatial planning and sustainable development in Nanjing.

## 1. Introduction

Cities are the key administrative units of land-use planning at home and abroad, and ecosystem service (ES) valuation plays a supporting role in urban land-use planning [1,2]. China has entered a new growth stage, with a dominating style of now being an urban society compared to a rural society in the past; thus, this new urbanization has become a national strategy and urban quality has been highly valued [3,4]. Yet, the rapid development of cities is also facing severe challenges, such as the continuous occupation of ecological space, low quality and function of ecosystem services (ESs), and continuous threats to biodiversity. Economic development and eco-environmental protection are highly conflicted [5,6,7].

Ecological conservation red line (ECRL) means the minimum spatial scope of ecological protection and the bottom line of ecological security, as well as the baseline area for the management of regional resources and the environment [8]. It is mainly delineated in important eco-function areas, ecologically fragile hotspots, and important species habitats, providing ESs to guarantee and maintain ecological security, living environment security, and biological security [9,10,11]. Although the concept of ECRL has not been clearly put forward internationally, species and the eco-environment are generally protected through the establishment of protected areas [12,13,14]. Studying China’s ECRL in preserving nature, biodiversity, and ESs has important reference value for other countries in the world, especially developing countries [10]. The ecological redline system was elevated to a national strategy at the Third Plenary Session of the 18th National Congress of the Communist Party of China in 2013, and in 2014, the Ecological Redline Policy (ERP) was officially incorporated into China’s Environmental Protection Law, which designated lands (ECRL) for strict protection to ensure sustainable provision of ESs. It aimed to use spatial planning to protect the ecological redline areas (ERAs), which has the potential to alleviate the contradiction between urbanization and shortage of ecological resources [15]. Once the ECRL has been demarcated, it should meet the management and control requirements of “no reduction in function and area”. Therefore, carrying out an ES valuation of the ERAs can effectively grasp the evolution process of ESs and their values, to evaluate the implementation effect of the ERP, with a view to optimize the spatial layout and analyzing the eco-environment effects during the period [16,17,18].

Current studies focusing on the ecosystem service value (ESV) variations of the regional ERAs over time have obtained relatively abundant academic results, indicating that ESs increase significantly under the ERP [9]. Moreover, some argue that the urbanization rate has a great impact on the values [19], and others have discussed the minimum protection area of the ECRL and the bottom line of ecological security [20]. However, the abovementioned research has two key limitations. First, it lacks a quantitative analysis of ERP implementation effectiveness. Second, the relevant policy recommendations for the regional land space planning by evaluating the ESV behind the spatial pattern dynamics in the ERAs have not been previously put forward.

Production–living–ecological space (PLES) was formally established as the national land classification method in the 18th National Congress of the Chinese Communist Party in 2012. Among them, (1) production space mainly provides agricultural products and services to ensure food security; (2) living space mainly provides industrial products and residential services; and (3) ecological space mainly provides ecological products and services to ensure ecological safety [21,22,23]. With the rapid economic growth and accelerated urbanization in China, production space, living space, and ecological space squeezed each other and the contradictions between them became increasingly acute [24,25]. Fierce competition for land use leads to major changes in the spatial layout of the country, causing dramatic fluctuations in ESV and ecological negative effects in some regions [26,27,28]. Not only are ecosystem services a carrier of spatial planning to shape the value of natural resources, but they are a government choice for spatial planning to promote public welfare as well [29]. Based on the spatial statistical analysis (PLES), exploring the spatial pattern and dynamic evolution of ESV is more conducive to vividly displaying the spatio-temporal evolution characteristics of ESV and grasping the spatio-temporal distribution law [30]. Analyzing the impact of PLES changes on ESV in a specific region, the essence of which is to classify all land-use types in the study area into three categories, would help to analyze the effect of land-use function transformation on ESV. Instead of the direct evaluation of land-use type changes, the study on ESV dynamics with the PLES classification method is more conducive to optimize the territorial spatial pattern and improve land-use efficiency by adjusting the spatial structure. Regarding urban dynamic changes of ESV based on PLES evolution, there are few relevant studies and the research scope is limited to specific areas, which does not involve the background of delineating and strictly abiding by the ECRL [31,32].

Assessing how the spatial pattern has changed over time and assessing the influence of these dynamics on ESV under the strategy of urban ECRL will provide decision-making references for regional management and control of ecological space and maintenance of ecological security, in particular, for cities with prominent contradictions between eco-social development and resource-environment protection. Nanjing, the only megacity in the Yangtze River Delta agglomeration, has seen rapid urbanization and the loss of ecological land due to continuous expansion of construction land in recent years. The Nanjing Ecological Civilization Construction Plan 2018–2020 (Revised and Compiled) clarifies that “one of the seven key tasks” of ecological civilization construction is to strictly observe the ECRL as the key point to build a PLES coordinated with the economy, resources, and environment. Therefore, it is of great practical significance to learn the evolution characteristics of the ESV caused by the PLES pattern transformation in the ERAs of Nanjing, which is conducive to promoting coordinated regional development.

On the basis of a PLES-perspective analysis, in this paper we (1) calculate the spatial structure and ESV dynamics over time, as well as the land-use transition affecting regional ESV at the ecological and non-ecological redline scale; (2) explore the gains and losses in ESV under PLES variation features in the ERAs and NERAs; (3) discuss the ERP effectiveness by horizontally comparing the response law of the spatial pattern and eco-environment; and (4) propose corresponding suggestions on land-use planning and spatial layout adjustment to improve regional ESV qualitatively, and further, to provide a theoretical basis for building a new pattern of territorial space development and protection in Nanjing. As such, it is hoped to better put the urban redline areas protection into practice from the map to the ground.

## 2. Materials and Methods

### 2.1. Study Area and Data Processing

Nanjing (31°14′ N~32°37′ N, 118°22′ E~119°14′ E), standing on the bank of the Yangtze River and guarding the Beijing–Shanghai artery, is not only the central city in the north wing of the Yangtze River Delta but also an important gateway that radiates to the development of the midwestern area. It is known as “the gateway to the south and east, the throat to the north and south ”, and there are 6 municipal districts, including Nanjing urban area, Jiangning District, Lishui District, Pukou District, Luhe District, and Gaochun District. In 2018, Nanjing’s GDP reached 1282.04 billion yuan, an increase of 8 percent, ranking the third among 16 cities with a GDP exceeding one trillion yuan. At the end of the year, the urban population was about 6.96 million, with a permanent urbanization rate of 82.50%. The per capita disposable income of urban and rural residents in the city was 52,916 yuan, an increase of 9.1 percent. The income ratio of urban and rural residents was 2.35:1, and the income gap continued to narrow.

Its geomorphic feature belongs to the Nanjing–Zhenjiang–Yangzhou hilly area, dominated by low mountains, hills, and downlands (60.8%), as well as plains, depressions, rivers, and lakes accounting for about 39.2%. The soil types are mainly divided into zonal soil and cultivated soil. Its soil distribution shows a certain regularity with topographic fluctuation and hydrological conditions, and can be divided into three categories: low mountain and hilly area, hillock area, and plain area. Nanjing has complex vegetation types, mainly including coniferous forest, deciduous broad-leaved forest, deciduous and evergreen broad-leaved mixed forest, bamboo forest, shrub, grass, and aquatic vegetation. The region is rich in water resources, and its water area covers 11% of the land area. Water area ecological land has a high ESV and is of great significance to the maintenance of biodiversity, the dominant functions of which include industrial water, irrigation, domestic water, fishery water, and so on. With the rapid development of the social economy and urbanization in Nanjing, urban construction land expands rapidly to the west, south, and east around the urban area of Nanjing.

**Figure 1:**
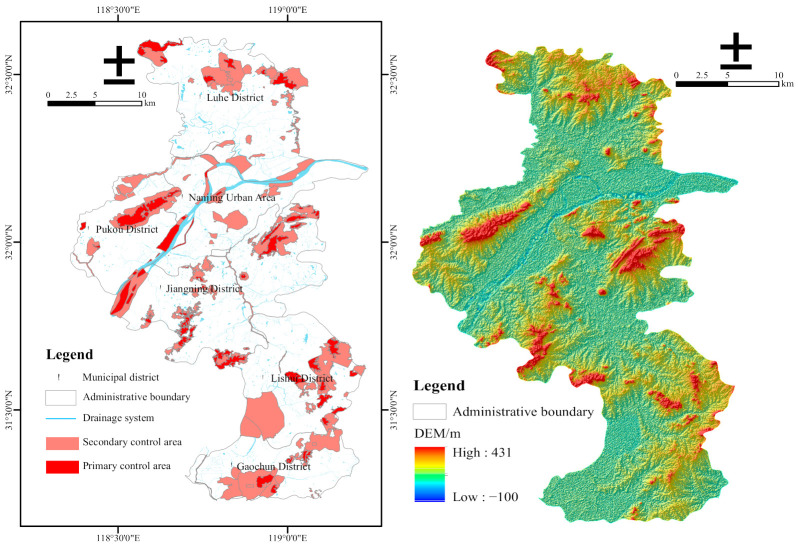
Regional distribution of the ecological red line and DEM rendering effect in Nanjing City.

Nanjing City covers nearly 6587 km^2^, where 93 ecological protection areas are delineated, divided into 12 different types with a total area of 1455.04 km^2^ (excluding the overlap). ERAs are managed at different levels, which can be divided into primary and secondary control areas. The primary control areas are the core of the ERAs, implementing the strictest control measures and strictly prohibiting all forms of development and construction activities. The secondary control areas focus on ecological protection, implement differentiated control measures, and prohibit the development and construction activities that damage the dominant ecological function. Among them, the primary control area covers 372.61 km^2^ and the secondary control area covers 1082.43 km^2^, accounting for 5.66% and 16.43% of the land area of the city, respectively. Part of the ERAs located outside the boundary of Nanjing was removed in GIS and then the sum area ultimately decreases to about 1440.71 km^2^. Using GIS and vector data of the ECRL planning to calculate the distribution and schematic diagram of the ERAs in Nanjing, we rendered Figure 1.

**Figure 2:**
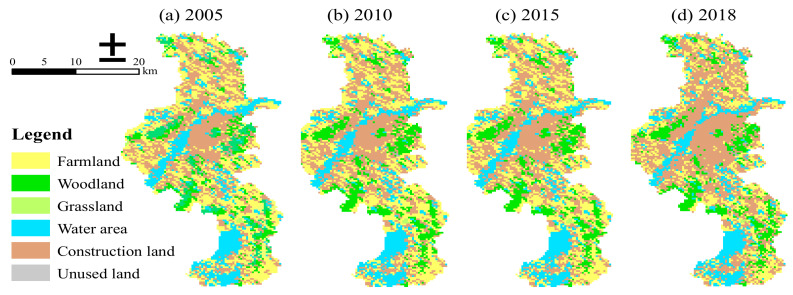
Land-use classification of Nanjing in (**a**) 2005, (**b**) 2010, (**c**) 2015, and (**d**) 2018.

Spatial and socioeconomic data from multiple sources were incorporated in the analysis of spatial patterns and ESV evolution over time. The ECRL planning vector data come from the Nanjing Eco-environment Bureau, and the DEM data (SRTM 90 m) were from the Resource and Environment Science and Data Center. The land-use data in 2005, 2010, 2015, and 2018 were interpreted from Landsat TM/ETM and Landsat 8 images (1 km × 1 km) with the pretreatment of geometric correction, radiation correction, and atmospheric correction (United States Geological Survey, Reston, VA, USA). A first-level classification system (farmland, woodland, grassland, water area, construction land, and unused land) was adopted in this paper processed with image clipping and reclassification in ArcGis (Esri, Redlands, CA, USA) and Envi (Exelis Visual Information Solutions, Boulder, CO, USA) tools (Figure 2). The areas planted wheat, paddy, and corn, and the grain yield per unit area and average price were from 2005–2018. The data were obtained from the Nanjing Statistical Yearbook and Compilation of Cost–Benefit Data of the National Agricultural Products [33,34]. Furthermore, the national grain consumer price index (CPI) was from the National Bureau of Statistics [35].

### 2.2. Ecosystem Service Valuation

ESs refer to the necessary ecological products and services provided by the ecosystem directly or indirectly to meet the needs of human survival, health, and well-being [36]. There is no unified evaluation system for ESs valuation, as the equivalent factor method and functional value method are two widely used ESs valuation methods. Based on distinguishing different ES functions, the former constructs the equivalent value of the different ESs, and then combined with the distribution area of each ecosystem to evaluate [26,36,37,38]. Besides, the value calculated by the latter depends on the amount and unit price of the functional quantity, and it is difficult to unify the evaluation method and parameter standard of each service value in this method [39,40]. In comparison with the functional value method, the equivalence factor method is more intuitive and requires less data, especially suitable for the assessment of ESV at regional scales [26,41]. The following formula was used to calculate the total value of the ecosystem services with all land-use types in the ecological and non-ecological redline scale of Nanjing: $ESV=\sum_{j=1}^{n}K_{j}P_{j}$, where $K_{j}$ is the ESV coefficient per land-use type, and $P_{j}$ is the coverage area per land-use type. Moreover, this paper also intends to make the corresponding adjustment planning for future territorial spatial structure by analyzing the impact of the PLES pattern evolution on ESV in the ERAs. The formula $R_{ij}=\left(K_{j}-K_{i}\right)\;\times \;A_{ij}$ was applied to calculate the gains and losses of ESV based on the transition from the initial-stage land-use type i into the final-stage land-use type j, where $K_{i}$ and $K_{j}$ stand for the ESV coefficient of the ith and jth land-use type, respectively; $A_{ij}$ presents the area where the *i*th land-use type is converted to the *j*th land-use type.

### 2.3. Quantification of ESs for Land-Use Types

To calculate the regional ESV depending on land coverage change over time, we used the ESs equivalent value per unit area table revised and supplemented to reclassify the land-use types [38]. The method divided ESs into four categories: supply services, regulation services, cultural services, and support services, with reference to the Millennium Ecosystem Assessment (MA) [42]. Based on the classification of China’s terrestrial ecosystem by Xie et al. in 2015 (Supplementary Table S1) and the land-use characteristics in the study area, the land-use types in Nanjing were divided into six categories: farmland, woodland, grassland, water area, construction land, and unused land. Among them, the land-use type classification was done by following several principles: the equivalent value of farmland was taken as the mean value of the paddy field and dryland; woodland equivalent value was the average value of coniferous, needle-broadleaf, broadleaf, and shrub; the ESs equivalent value of grassland, water area, and unused land corresponded to the shrubbery, water system, and bare land, respectively; and then the ESV of the construction land was considered as zero [36]. The revised ecosystem service equivalent value per unit area is shown in Table 1.

### 2.4. Evaluation of PLES Structure Change

In recent years, the optimization of domestic territorial space tends to the planning framework system led by PLES [43]. In this paper, the territorial space is divided into production space (farmland), living space (construction land), and ecological space (woodland, grassland, water area, and unused land) [23,44]. Among them, firstly, the production space principal offers agricultural products and services. Secondly, the leading function of the living space is to provide human habitation, consumption, leisure, and entertainment. Thirdly, the ecological space mainly provides ecological products and services, and plays an important role in the regulation, maintenance, and protection of regional ecological security [43]. As the demarcated area of the ERAs and NERAs is fixed, according to the four phases of land-use data interpreted by remote sensing, the distribution and structure evolution characteristics of PLES in the study area from 2005 to 2018 were analyzed. Combining GIS tools to reclassify the land-use types of the ERAs and NERAs, we obtained the spatial distribution and structure change in PLES in the corresponding year.

### 2.5. Accounting and Revision of the Unit Equivalence Factor Value

Drawing on the 2016–2019 Nanjing Statistical Yearbook, it is known that the total output of wheat, rice, and corn in 2005, 2010, 2015, and 2018 accounted for more than 94% of the total grain output. As such, we regarded the abovementioned crops as the main grain products in the study area. Based on “the value of an equivalence factor is equal to 1/7 of the market value of grain per unit area in the current year” [37], we computed the economic value of the standard unit equivalence factor, in combination with the national average market price of the grain crops, which was manifested in the following formula: $E_{n}=\frac{1}{7}\sum_{m=1}^{n}\frac{s_{m}p_{m}q_{m}}{S}\left(m=1,\ldots ,n\right)$, where $E_{n}$ is the economic value of the food production service provided by the farmland ecosystem per unit area (yuan/ha), namely, the ESV coefficient before correction; m represents the main food crops, including wheat, rice, and corn; s is the planting area of regional crops (ha); p is the national average price (yuan·kg^−1^); q is the yield per unit area of grain (kg/ha); and S is the total area of food crops (ha). Data on the yield per unit area, average market price, and sown area of wheat, rice, and corn in Nanjing from 2005 to 2018 are shown in Table 2.

To eliminate the influence of natural factors and inflation on grain price fluctuations, this paper introduced the revised ESV coefficient of the national grain fixed-base consumer price index (CPI) [45], and the modified results are shown in Table 3.

## 3. Results

### 3.1. Evolution Characteristics of the PLES Pattern

**Figure 3:**
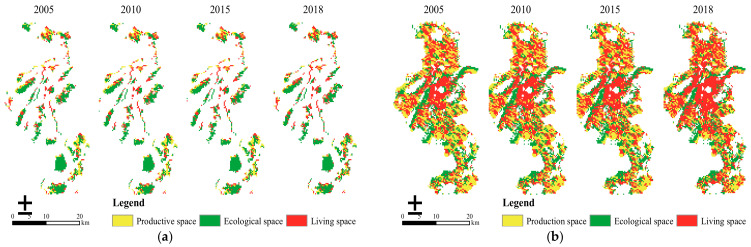
(**a**) Spatial distribution of PLES in ecological redline areas during 2005–2018; (**b**) spatial distribution of PLES in non-ecological redline areas during 2005–2018.

From the perspective of the PLES pattern distribution (Figure 3a), the ERAs in 2005, 2010, 2015, and 2018 were dominated by ecological space and distributed in the form of clumps, especially concentrated in the Jiangning District and Lishui District; production space was mainly distributed in the border zone of the northwest and southeast of the ERAs; and living space presented a dotted distribution and its patch area is small. Given the spatial quantity structure (Figure 3b), ecological space accounted for about 57% of the land area, and production space was less than a quarter of the total area; the proportion index of both was the highest and smallest of PLES in the ERAs, respectively. With the development of urban expansion and social economy, living space continued to grow and its area had increased by 67.47 km^2^ in 13 years, with an overall escalating rate of 24.71%; ecological space showed a tendency of first declining and then growing, with the area descending by 4.21 km^2^ from 2005 to 2015, and increasing by 22.02 km^2^ from 2015 to 2018, which reflected an overall increase of 2.43%; in turn, the production space had been decreasing year by year, where the area in the past 13 years had dropped by 83.69 km^2^ and the overall descent rate was 32.02%.

**Figure 4:**
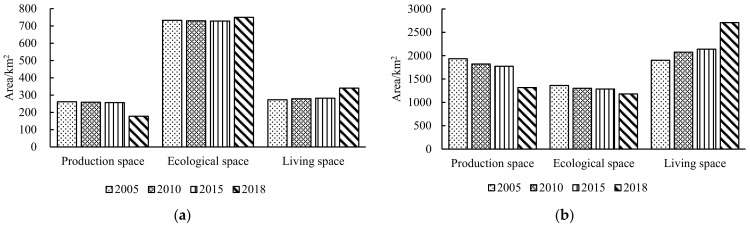
(**a**) Spatial structure change of PLES in ecological redline areas during 2005–2018; (**b**) spatial structure change of PLES in non-ecological redline areas during 2005–2018.

From the perspective of PLES pattern distribution (Figure 4a), in 2005, 2010, 2015, and 2018, the ecological space of the NERAs was mostly distributed in strips, occupying the smallest proportion of the space; production space was scattered and the patch area was large; and living space was centered in Nanjing Urban Area, forming an obvious agglomeration and gradually radiating outward. With the accelerated pace of urbanization and industrialization, production space and ecological space were continuously eroded during the study period and living space in the surrounding cities expanded rapidly, among which Jiangning District, Nanjing Urban Area, and Luhe District increased the most significantly. In view of the spatial quantity structure (Figure 4b), from 2005 to 2018, ecological space in the NERAs was largely occupied by production space and ecological space. In 2005, production space dominated the regional spatial pattern, while living space occupied a dominant position in 2010, 2015, and 2018, with the proportion of both remaining between 73% and 78% in each year. The overall variation tendency of PLES in the NERAs showed the characteristics of “one rise and two drops” from 2005 to 2018: living space presented an upward trend, and the proportion of which increased from 36.57% in 2005 to 52.02% in 2018, with an increase as high as 42.42%; the proportion of production space and ecological space significantly reduced, from 37.22% and 26.22% in 2005 to 25.30% and 22.69% in 2018, respectively, with a decrease of 31.94% and 13.36%.

In summary, rather than the minimum ecological space scale in the NERAs, ERAs gave high priority to ecological space (Table 4). Ecological space of the ERAs accounted for a larger proportion than the NERAs of the total land area, which proved ecological products and services in Nanjing were mainly provided by the ERAs to improve the urban eco-environment [46]. In recent years, the NERAs have shifted from being dominated by production space to living space, indicating a shift in the regional development center. During the rapid urbanization and industrialization process in Nanjing, the expansion of construction land in the ecological and non-ecological redline areas from 2005 to 2018 had been increasing continuously, and the proportion of living space showed a sustained increasing tendency, whereas production space and ecological space have been threatened.

### 3.2. The Value Analysis of Various ESs

Using the revised ESV coefficient table corresponding to different land-use types, combined with the area of each category in the period, the value of the individual ESs and of per unit area are finally obtained in the ERAs and NERAs from 2005 to 2018 (Table 5 and Table 6).

For the value of single ESs in the ERAs and NERAs, the value of the water regulation ES was up to more than 70% of the total value, and the value of the soil conservation ES accounted for less than 1%. From the perspective of the variations in individual ESs, only the value of the water supply ES in the two regions reflected a small increase in the past 13 years, increasing by 31 million yuan and 61 million yuan, respectively, while the value of the water regulation service both decreased the most, with losses of 172 million yuan and 2.509 billion yuan, respectively, accounting for 42.12% and 60.86% of the total value loss of all types of services. In view of the gradient of each service value in the different periods, the service value of the two regions fluctuated slightly in 2005–2010 and 2010–2015, and the overall trend was relatively flat, while the reduction rate in the individual value of the ESs in 2015–2018 were characterized by drastic fluctuations.

**Figure 5:**
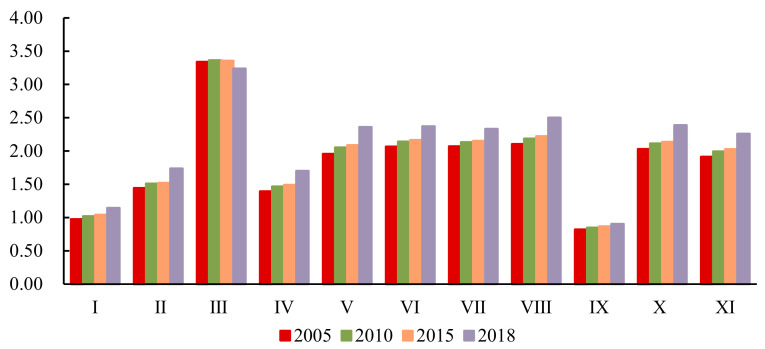
Proportion in ESs value per unit area between the ERAs and NERAs in 2005, 2010, 2015, and 2018. Ⅰ: Food production; Ⅱ: Raw material production; Ⅲ: Water supply; Ⅳ: Gas regulation; Ⅴ: Climate regulation; Ⅵ: Decontamination environment; Ⅶ: Water regulation; Ⅷ: Aesthetic landscape; Ⅸ: Soil maintenance; Ⅹ: Nutrient cycling; Ⅺ: Biodiversity protection.

Dividing the per unit area value of the NERAs by that of the ERAs in the corresponding year, the proportion obtained demonstrated the value created by various ESs per unit area in the two regions (Figure 5). Despite the ratio of water supply service reflecting a descending tendency, quite apart from that, other ESs still showed continual growth since 2005, especially gas regulation and climate regulation services. In comparison to the NERAs, the per unit area value of the soil maintenance service was slightly smaller in the ERAs. Conversely, for certain ESs, such as water supply, climate regulation, decontamination environment, water regulation, aesthetic landscape, nutrient cycling, and biodiversity protection in the ERAs, the per unit area value of each was approximately twice more than that in the NERAs. In particular, the water supply service was up to 3-fold that of the NERAs.

**Figure 6:**
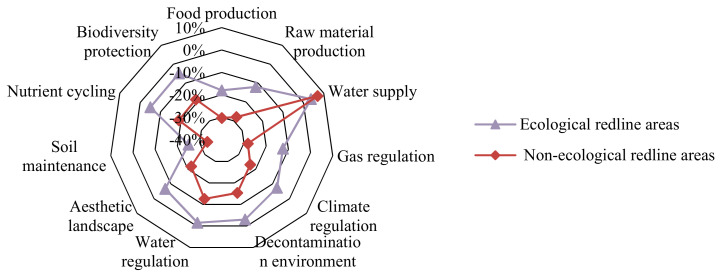
Change rate of ecosystem service value in ecological and non-ecological redline areas during 2005–2018.

According to the overall gradient of each ES (Figure 6), from 2005 to 2018, the value of each individual ES in the NERAs changed more significantly than that in the ERAs, and the total value of all types of services in the two regions decreased by 14.62 % and 2.56%, respectively; that is, the reduction in the NERAs in the past 13 years was close to 6-fold that of the ERAs, which fully illustrated the significance of delineating the ERAs. During 2005–2018, the soil maintenance service in the ERAs and NERAs showed a trend of rapid decline, with value dropping by 25.01% and 33.46%, respectively. Food production, raw material production, and gas regulation services all reflected a greater decline, with a decrease of above 12% and 25%, respectively; although the gradient of the four services—decontamination environment, aesthetic landscape, nutrient cycling, and biodiversity protection—were relatively low, the reduction rates of each in the ERAs and NERAs were also above 3% and 15%, respectively.

### 3.3. Roll-In and Roll-Out of Land-Use Types

The ESV in the ERAs and NERAs of Nanjing City dropped from 159.80 × 10^8^ RMB to 155.70 × 10^8^ RMB and 281.91 × 10^8^ RMB to 240.68 × 10^8^ RMB during the 13 years, respectively. A certain amount of loss in ESV was both reflected in the ecological and non-ecological redline scale during the period, essentially arising from the influence of the regional land-use transition directly on the PLES pattern that ultimately changed the ESV. For the analysis undertaken in this study, the years 2005 to 2018 were used to give a snapshot of land-use coverage change in the ERAs and NERAs. The land-use transition matrix, using GIS tools to analyze the variation quantity of each land-use type affecting the regional static values, is shown in Table 7 and Table 8 [47].

The results showed that all land-use types in the ERAs during 2005–2018 were mainly transferred to construction land, particularly with the conversion of farmland, woodland, and water area to construction land. Meanwhile, the contribution ratio of these three land-use types in the ERAs reached 62.01%, 18.04%, and 15.94%, respectively, regarding the increase of construction land; and 62.09%, 18.55% and 18.08%, respectively, in the NERAs.

From 2005 to 2018, there were roll-in and roll-out of land-use types to some extent in the ERAs and NERAs. Judging from the transfer-out ratio of various land types in 2005, with the exception of construction land and unused land, more than 3% and 20% of the remaining land-use types in the two regions were converted to construction land, and the proportion of farmland transferred was, respectively, up to 16.90% and 26.54%. In terms of various land-use sources in 2018, except for the unused land that was transferred from woodland, more than 80% and 60% of the remaining land-use types in the two regions were derived from the unchanged land in 2005. In addition, grassland, construction land, woodland, and water area in 2018 were mainly supplemented by farmland, of which the total area of construction land in the two regions covered 12.20% and 19.09% of the original cultivated land respectively.

As a consequence, the core transformation of land-use types in the ERAs and NERAs was the rapid expansion of construction land followed by higher-valued land types (farmland, woodland, and water area) turning into a lower-valued land type (construction land) in the period of 2005–2018.

### 3.4. Impact of Land-Use Transitions on ESV

Regional ecological quality frequently has two opposite tendencies at the same time—eco-environment improvement and deterioration—and both of them will offset each other to a large extent to make ecological quality remain relatively stable on the whole. However, it does not signify no change in the regional eco-environment, tending to appear as positive effects on eco-environment (PEE) > negative effects on eco-environment (NEE) [27,31] or PEE < NEE [32,48].

Based on the land-use transition matrix, to make corresponding adjustments to the territorial spatial layout, an analysis was carried out to explore how the specific land-use transitions and spatial structure changes have an adverse and favorable effect on ESV reduction and enhancement in the ERAs and NERAs, respectively (Table 9 and Table 10).

In terms of land-use transitions, for one thing, ESV reduction was due primarily to the decline in unit equivalence factor value from 2005 to 2018, especially since the water area ecosystem remains unchanged, which led to the value attenuation in the ERAs (655.20 × 10^6^ RMB) and NERAs (880.47 × 10^6^ RMB), with the contribution rate accounting for 43.37% and 11.31%, respectively. For another thing, with the acceleration of urbanization and industrial development, a large number of agricultural production land and ecological land have been squeezed, ultimately resulting in the spatial structure imbalance and environmental degradation in the two regions. Among them, the encroachment of construction land on farmland, woodland, and water area was the leading cause of the ESV decrease during 13 years, with the contribution rates accounted for 3.82%, 5.54%, and 31.20% in the ERAs, and 7.04%, 10.49%, and 65.22% in the NERAs, respectively. Besides, the conversion of farmland and woodland to water area was the greatest contributor to ESV increase in the ERAs (56.30% and 23.08%) and NERAs (71.35% and 10.53%).

### 3.5. Impact of PLES Pattern Changes on ESV

In view of the PLES pattern changes on ESV, the occupation of living space on ecological space made a big difference in the ESV reduction, with a contribution rate of 37.97% and 75.71% in the ERAs and NERAs, respectively. The primary reason for the ESV enhancement in the ERAs in the study period was the conversion of production space to ecological space and the transformation of eco-lands within ecological space. The former had the highest contribution index, increasing the ESV, and the same was true for the NERAs, with a total contribution ratio of 66.06% and 77.27%, respectively, which illustrated the significance of returning farmland to woodland and water area. Among them, the conversion of farmland to water area contributed most to improving the ESV, with a contribution ratio of 56.30% and 71.35%. Besides, the latter dedicated 30.57% to the value added. By contrast, in the NERAs, the transformation from living space to ecological space increased the ESV by 272.57 × 10^6^ RMB, with a low contribution ratio of 7.74%, largely due to the need for urban expansion and economic development resulting in a limited decline.

**Figure 7:**
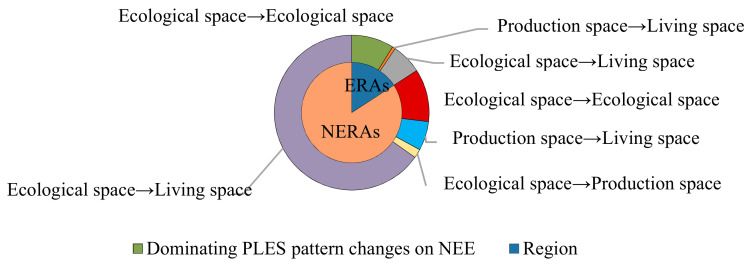
Ratio of dominating PLES pattern changes on NEE in the ERAs and NERAs.

**Figure 8:**
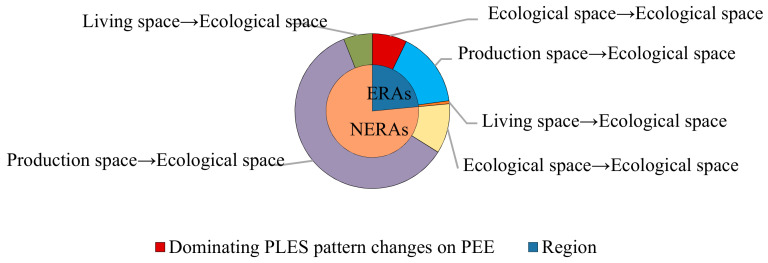
The ratio of dominating PLES pattern changes on PEE in the ERAs and NERAs.

In addition, notwithstanding the similar dominating PLES pattern changes on NEE and PEE, the ratio between ERAs and NERAs was quite different (Figure 7 and Figure 8). The negative effects on regional eco-environment in the ERAs and NERAs were estimated to be 15.85% and 84.15% of terrestrial impairment, and the positives of terrestrial appreciation accounted for 23.49% and 76.51%, respectively. The fact is that deterioration and improvement of regional ecological quality had been disproportionate in the ecological and non-ecological redline regions, in which the value reduction could be primarily attributed to eco-lands remaining unchanged in the ecological space (55.92% of ERAs) and ecological space turning to living space (77.50% of NERAs), while value growth was mainly due to the conversion of production space to ecological space (66.54% of ERAs, and 78.54% of NERAs).

## 4. Discussion

### 4.1. The Validity of ERP

The ERAs and NERAs of Nanjing cover an area of 1440.71 km^2^ and 5146.29 km^2^, respectively, and the ratio of the two regions is close to 1:3.6. The reduction in ESV in the ERAs and NERAs of Nanjing City is estimated to be 4.09 × 10^8^ RMB and 41.23 × 10^8^ RMB between 2005 and 2018, respectively, with a decrease of 2.56% and 14.63%, thereby proving that a certain amount of damage to the ESV was mirrored in the two regions during the period.

Nevertheless, from the perspective of the ESV per unit area, the value of the ERAs in each year was 110.92 × 10^3^ RMB/ha, 123.75 × 10^3^ RMB/ha, 118.38 × 10^3^ RMB/ha, and 108.08 × 10^3^ RMB/ha, while of the NERAs was 54.78 × 10^3^ RMB/ha, 59.11 × 10^3^ RMB/ha, 56 × 10^3^ RMB/ha, and 46.77 × 10^3^ RMB/ha. In other words, the ESV per unit area created by the ERAs was about twice that of the NERAs, which fully illustrated the significance of the ERP effectiveness.

From the perspective of the change amount and rate of ESV, the value of the ESs in the ERAs and NERAs decreased to varying degrees from 2005 to 2018, reducing by 4.09 × 10^8^ yuan and 41.23 × 10^8^ yuan, respectively, a decrease of 2.56% and 14.62%. The depreciation of the ESV in the NERAs is close to 10 times that of the ERAs, and the decreasing extent of which is close to 6 times that of the ERAs, which fully illustrated that delineating the ECRL can effectively slow down the trend of eco-environment deterioration.

By comparison, the value loss per unit area was 0.99 × 10^6^ RMB and 1.48 × 10^6^ RMB for the ERAs and NERAs, respectively; conversely, the average value addition was 0.74 × 10^6^ RMB/km^2^ and 0.67 × 10^6^ RMB/km^2^, demonstrating that the ecological deterioration was more serious in the NERAs.

### 4.2. Land-Use Planning and PLES Structural Adjustment

By horizontally comparing with the NERAs, it is concluded that ECRL delineation is conducive to the establishment of an ecological security pattern, and is further beneficial to highlight the importance and necessity of improving ESV in the ERAs. As a consequence, by exploring the major transformation of land-use types and spatial structure variations affecting eco-environment quality, this study aimed to propose land-use planning and PLES structural adjustments, to guide the future territorial space management and control of Nanjing.

In totality, from the perspective of specific land-use transition, the process of eco-response, reasonably increasing such eco-lands as woodland and water area and weakening the disturbance of human activities, benefited the eco-environment in the ERAs. Thus, it indicated that the terrestrial realm of Nanjing should actively implement the policies of returning farmland to woodland and water area, slowing down the expansion of construction land and promoting the conversion of other land-use types to water area, with a view to improving the ESV.

In this paper, despite the eco-environment quality having declined slightly in both the ERAs and NERAs (PEE < NEE), the dominating PLES pattern changes on PEE and NEE varied significantly in the two regions, and the specific transition of land-use types were not taken into account. To adjust the territorial spatial structure, promoting the transition from production space to ecological space and depressing the encroachment of living space on other spaces, was instrumental in releasing the decline of the eco-condition. In general, guaranteeing production space, encouraging the transition from living space to ecological space, and increasing the proportion of ecological space are conducive to building a spatial pattern that is coordinated among the production, living, and ecological spaces in Nanjing.

### 4.3. Limitations and Future Research

Our analyses have several key limitations. First, our correction of the ESV coefficient adopted the equivalence factor method, and so does not account for the ecosystem quality status; thus, the net primary production and vegetation coverage could be combined [18,32,49]. The trade-offs within ESs were not addressed in this paper, but these issues of structure and diversity can be further explored in future research.

We also focused on the assessment of six land-use types, yet in essence, a second-level classification will be needed to develop the appropriate regional land-use planning, subdividing into each second-level land-use type regulation. Furthermore, the importance of effective land-use planning for a reduction in earthquake catastrophe was highly stressed [50], and future analyses could calculate the numerical value of increasing or decreasing land-use area [51]. Although our endeavors to explore PLES structural adjustment could better benefit territorial spatial governance, suggestions in this paper remained derived from qualitative analysis, and there were no clear visual results to solve the issue. Blindly increasing or reducing a certain space was more likely to cause a serious imbalance between them, thereby what matters was that we could adjust the proportion of production, living, and ecological spaces quantitatively to increase the ESV in the research area. Integrating with the current spatial proportion of space types in the ERAs and NERAs, additional research on the identification of thresholds is needed to determine the minimum space scope of ecological protection, in consideration of the complexity, vulnerability, and stability of PLES [52].

## 5. Conclusions

In light of the above analysis and discussion, this paper summarized the research results from the following two aspects: (1) we took the ERAs as the research object, on the one hand, with a horizontal comparison of the evolution characteristics of the ESs and their value in the NERAs; and (2) on the other hand, with a temporal analysis of the cause of each one’s ESV reduction and the countermeasures for spatial adjustment. Based on the above, the following conclusions can be drawn:

### 5.1. Horizontal Comparison on the ERAs and NERAs

With the rapid progress of urbanization, the proportion of living space in the ERAs and NERAs has both been continuously increasing in the past 13 years, and the PLES structure has gradually become unbalanced, which threatens production space and ecological space. The overall variation trend of the ESV in the two regions from 2005 to 2018 was consistent. From 2005 to 2010, ESV fluctuated and increased, and the value impairment from 2010 to 2018 was significantly higher than that in the previous five years. Water regulation service was the leading function of the two regions. Soil maintenance service decreased the most, and the value of which per unit area in the ERAs was less than that in the NERAs, namely, it is essential for the ERAs to give priority to improving the soil conservation service function to enhance the ability of resisting flood and waterlogging disaster and preventing soil degradation.

Furthermore, the proportion index of ecological space in the ERAs was the highest, while it was the lowest in the NERAs; the reduction in ESV in the ERAs was significantly smaller than the NERAs; and the per unit area value of the ESs in the ERAs was approximately twice than that in the NERAs, which fully illustrated the ERP effectiveness.

### 5.2. Temporal Analysis of the Evolution of ESV and Spatial Structure

With the urban expansion and social-economic development, living space continues to grow, ecological space shows a trend of decline as a whole, and production space had been decreasing year by year in Nanjing.

The ESV of Nanjing showed a descending tendency in 2005–2018, and the negative effects of the eco-environment in the study area were greater than the positives. From the perspective of specific land-use transformation modes, the main reason for the reduction of ESV is the encroachment of farmland, woodland, and water area by construction land; the main reason for the increase of ESV was the conversion of farmland to woodland and water area. From the perspective of spatial structure transition, the main cause of the ESV reduction was the occupation of ecological space by living space; the main cause of the ESV enhancement was that the production space and living space turned to ecological space.

In conclusion, it is necessary for us to focus on protecting ecological lands such as woodland, grassland, and water area. We should also strictly control the total amount and intensity of construction land, promoting its development from a sprawl to a compact type. Besides, given the spatial structural adjustment, we should restrict living space from occupying ecological space, ensure and intensively use production space to improve the economic benefits of production land, and encourage the transformation of living space into ecological space. Ecological restoration does have an impact on the changes in land-use types and landscape patterns at a significant level and positively affected the ESV. There are many methods and approaches for ecological restoration. Our paper adopts “territorial space ecological restoration”, which is to improve the ESV through territorial spatial pattern planning. To sum up, this research is an attempt to promote the sustainable development of the economy and social ecology in Nanjing.

## Figures and Tables

**Table 1:** Revised ecosystem service equivalent value per unit area.

Ecosystem Classification		Farmland	Woodland	Grassland	Water Area	Unused Land
Supply services	Food production	1.105	0.2525	0.38	0.80	0
	Raw material production	0.245	0.5800	0.56	0.23	0
	Water supply	−1.305	0.3000	0.31	8.29	0
Regulation services	Gas regulation	0.890	1.9075	1.97	0.77	0.02
	Climate regulation	0.465	5.7075	5.21	2.29	0
	Decontamination environment	0.135	1.6725	1.72	5.55	0.1
	Water regulation	1.495	3.7350	3.82	102.24	0.03
Cultural services	Aesthetic landscape	0.075	0.9275	0.96	1.89	0.01
Support services	Soil maintenance	0.520	2.3225	2.40	0.93	0.02
	Nutrient cycling	0.155	0.1775	0.18	0.07	0
	Biodiversity protection	0.170	2.1150	2.18	2.55	0.02

**Table 2:** Grain yield, price, and sowing area of Nanjing in 2005, 2010, 2015, and 2018.

Year	Grain Yield/kg·ha^−1^			Price/Yuan·kg^−1^			Sowing Area/10^3^ ha		
	Wheat	Paddy	Corn	Wheat	Paddy	Corn	Wheat	Paddy	Corn
2005	3934	7424	4692	1.3802	1.5532	1.1106	18.60	108.19	7.99
2010	4694	8189	6761	1.9802	2.3600	1.8724	44.92	96.63	8.51
2015	5257	8733	6375	2.3286	2.7604	1.8846	45.91	91.33	8.90
2018	4911	8670	6507	2.2436	2.5884	1.7560	53.14	86.10	4.85

**Table 3:** The correction result of the unit equivalence factor value.

Year	Unit Yield/ (Yuan·ha^−1^)	Unit Equivalence Factor Value/Yuan	National Grain CPI (2005 = 100)	Revised Unit Equivalence Factor Value/Yuan
2005	10,314.31	1473.47	100	2681.863
2010	19,148.26	2277.89	137.91	3006.299
2015	23,447.33	2806.10	177.01	2885.364
2018	20,378.25	2551.12	182.01	2551.120

Abbreviations: CPI: Consumer Price Index.

**Table 4:** The proportion of production, ecological, and living spaces in the ERAs and NERAs between 2005 and 2018.

Year	ERAs			NERAs		
	Production Space	Ecological Space	Living Space	Production Space	Ecological Space	Living Space
2005	0.36	1	0.37	1.42	1	1.39
2010	0.35	1	0.38	1.40	1	1.59
2015	0.35	1	0.39	1.38	1	1.66
2018	0.24	1	0.45	1.12	1	2.29

Abbreviations: ERAs: Ecological Redline Areas, NERAs: Non-ecological Redline Areas.

**Table 5:** Ecosystem service value in ecological and non-ecological redline areas during 2005–2018 (10^8^ RMB).

First Classification	Second Classification	ERAs				NERAs			
		2005	2010	2015	2018	2005	2010	2015	2018
Supply services	Food production	2.09	2.32	2.22	1.72	7.67	8.13	7.63	5.35
	Raw material production	1.08	1.19	1.14	0.95	2.68	2.84	2.67	1.94
	Water supply	8.13	9.10	8.70	8.44	8.71	9.66	9.26	9.32
Regulation services	Gas regulation	3.63	4.03	3.86	3.18	9.31	9.84	9.26	6.68
	Climate regulation	8.91	9.90	9.49	8.25	16.26	17.23	16.28	12.49
	Decontamination environment	7.88	8.79	8.41	7.65	13.62	14.67	13.9	11.53
	Water regulation	115.51	128.97	123.33	113.78	199.52	216.12	204.9	174.43
Cultural services	Aesthetic landscape	3.54	3.94	3.78	3.31	6.04	6.41	6.06	4.72
Support services	Soil maintenance	0.71	0.79	0.75	0.53	3.15	3.32	3.11	2.10
	Nutrient cycling	5.14	5.72	5.48	4.88	9.04	9.67	9.15	7.33
	Biodiversity protection	3.17	3.53	3.38	3.02	5.90	6.32	5.98	4.80
Total		159.80	178.28	170.55	155.70	281.91	304.19	288.20	240.68

**Table 6:** Per unit area value in ecological and non-ecological redline areas during 2005–2018 (10^3^ RMB/ha).

First Classification	Second Classification	ERAs				NERAs			
		2005	2010	2015	2018	2005	2010	2015	2018
Supply services	Food production	1.45	1.61	1.54	1.19	1.49	1.58	1.48	1.04
	Raw material production	0.75	0.83	0.79	0.66	0.52	0.55	0.52	0.38
	Water supply	5.64	6.32	6.04	5.86	1.69	1.88	1.80	1.81
Regulation services	Gas regulation	2.52	2.8	2.68	2.21	1.81	1.91	1.80	1.30
	Climate regulation	6.18	6.87	6.59	5.73	3.16	3.35	3.16	2.43
	Decontamination environment	5.47	6.1	5.84	5.31	2.65	2.85	2.70	2.24
	Water regulation	80.17	89.52	85.6	78.98	38.77	41.99	39.82	33.89
Cultural services	Aesthetic landscape	2.46	2.73	2.62	2.3	1.17	1.25	1.18	0.92
Support services	Soil maintenance	0.5	0.55	0.52	0.37	0.61	0.65	0.60	0.41
	Nutrient cycling	3.57	3.97	3.8	3.39	1.76	1.88	1.78	1.42
	Biodiversity protection	2.2	2.45	2.35	2.1	1.15	1.23	1.16	0.93
Total		110.92	123.75	118.38	108.08	54.78	59.11	56.00	46.77

**Table 7:** Land-use transition matrix in ecological redline areas during 2005–2018 (ha).

	Grassland	Farmland	Construction Land	Woodland	Water Area	Unused Land
Grassland	1710.12	0	351.58	0	300	0
Farmland	100	22,068.60	5442.15	2636.89	1946.92	0
Construction land	0	87.20	35,814.88	55.29	88.64	80.40
Woodland	64.16	62.38	1583.61	37,218.87	923.97	950.94
Water area	0	1.15	1399.20	3.08	39,896.11	3.08
Unused land	0	0	0	0	0	400

**Table 8:** Land-use transition matrix in non-ecological redline areas during 2005–2018 (ha).

	Grassland	Farmland	Construction Land	Woodland	Water Area	Unused Land
Grassland	2478.30	38.98	1024.32	0	374.71	0
Farmland	341.63	129,510.83	51,734.39	5261.20	8111.15	0
Construction land	0	2313.50	187,710.78	397.46	850.58	100
Woodland	306.16	591.53	15,456.27	44,840.50	1386.33	467.12
Water area	0	510.87	15,065.71	0	53,613.51	0
Unused land	0	0	39.04	0	0	660.96

**Table 9:** PLES pattern changes affecting ESV in the ERAs during 2005–2018. A contribution rate <1 was not under consideration.

	PLES Pattern Changes	Land-Use Transitions	ESV Variations/10^6^ RMB	Contribution Rate/%
NEE	Ecological space→Ecological space	remaining woodland	−95.85	6.35
		remaining water area	−655.20	43.37
		woodland→unused land	−49.75	3.29
		total	−800.80	53.01
	Production space→Living space	farmland→construction land	−57.65	3.82
	Ecological space→Living space	grassland→construction land	−18.57	1.23
		woodland→construction land	−83.66	5.54
		water area→construction land	−471.35	31.20
		total	−573.58	37.97
	Total		−1432.03	94.80
PEE	Ecological space→Ecological space	grassland→water area	80.29	7.49
		woodland→water area	247.27	23.08
		total	327.56	30.57
	Production space→Ecological space	farmland→woodland	104.57	9.76
		farmland→water area	603.26	56.30
		total	707.83	66.06
	Living space→Ecological space	construction land→water area	28.40	2.65
	Total		1063.79	99.28

Abbreviations: NEE: Negative Effects on Eco-environment, PEE: Positive Effects on Eco-environment.

**Table 10:** PLES pattern changes affecting ESV in the NERAs during 2005–2018. A contribution rate < 1 was not under consideration.

	PLES Pattern Changes	Land-Use Transitions	ESV Variations/10^6^ RMB	Contribution Rate/%
NEE	Ecological space→Ecological space	remaining woodland	−115.48	1.48
		remaining water area	−880.47	11.31
		total	−995.95	12.80
	Production space→Living space	farmland→construction land	−548.04	7.04
	Ecological space→Production space	water area→farmland	−166.95	2.15
	Ecological space→Living space	woodland→construction land	−816.49	10.49
		water area→construction land	−5075.17	65.22
		total	−5891.66	75.71
	Total		−7602.60	97.69
PEE	Ecological space→Ecological space	grassland→water area	100.29	2.85
		woodland→water area	371.01	10.53
		total	471.30	13.38
	Production space→Ecological space	farmland→woodland	208.65	5.92
		farmland→water area	2513.26	71.35
		total	2721.91	77.27
	Living space→Ecological space	construction land→water area	272.57	7.74
	Total		3465.77	98.39

## Data Availability

The data presented in this study are available on request from the corresponding author.

## Supplementary Materials

The following are available online at https://www.mdpi.com/article/10.3390/ijerph18084224/s1, Table S1: The equivalents of ESV supplied by per unit area of ecosystem.
