# Supplementary material for: Spatial Pattern Change and Ecosystem Service Value Dynamics of Ecological and Non-Ecological Redline Areas in Nanjing, China
Source: Int J Environ Res Public Health. 2021 Apr 16;18(8):4224. doi: 10.3390/ijerph18084224 (PMC8073379; doi:10.3390/ijerph18084224)
Supplement: Supplementary file 1 [file ijerph-18-04224-s001.zip › ijerph-1160413-supplementary.pdf]

Supplementary Table S1. The equivalents of ESV supplied by per unit area of ecosystem [38]

**Ecosystem classification**		**Supply services**			**Regulation services**				**Support services**			**Cultural services**
Primary	Secondary	Food production	Raw material production	Water supply	Gas regulation	Climate regulation	Decontamination environment	Water regulation	Soil maintenance	Nutrient cycling	Biodiversity protection	Aesthetic and landscape
Farmland	Dry land	0.85	0.4	0.02	0.67	0.36	0.1	0.27	1.03	0.12	0.13	0.06
	Paddy field	1.36	0.09	-2.63	1.11	0.57	0.17	2.72	0.01	0.19	0.21	0.09
Woodland	Coniferous	0.22	0.52	0.27	1.7	5.07	1.49	3.34	2.06	0.16	1.88	0.82
	Mixed	0.31	0.71	0.37	2.35	7.03	1.99	3.51	2.86	0.22	2.6	1.14
	Broad-leaved	0.29	0.66	0.34	2.17	6.5	1.93	4.74	2.65	0.2	2.41	1.06
	Bush	0.19	0.43	0.22	1.41	4.23	1.28	3.35	1.72	0.13	1.57	0.69
Grassland	Prairie	0.1	0.14	0.08	0.51	1.34	0.44	0.98	0.62	0.05	0.56	0.25
	Shrub	0.38	0.56	0.31	1.97	5.21	1.72	3.82	2.4	0.18	2.18	0.96
	grass	0.22	0.33	0.18	1.14	3.02	1	2.21	1.39	0.11	1.27	0.56
Wetland	Wetland	0.51	0.5	2.59	1.9	3.6	3.6	24.23	2.31	0.18	7.87	4.73
Bareland	Desert	0.01	0.03	0.02	0.11	0.1	0.31	0.21	0.13	0.01	0.12	0.05
	Barren	0	0	0	0.02	0	0.1	0.03	0.02	0	0.02	0.01
Water area	Water	0.8	0.23	8.29	0.77	2.29	5.55	102.24	0.93	0.07	2.55	1.89
	Glacier and snow	0	0	2.16	0.18	0.54	0.16	7.13	0	0	0.01	0.09
